# TREM‐1 Blockade Inhibits Inflammasome Activation and Pyroptosis: Novel Insights on the Role of TREM‐1 and Syk in Monosodium Urate Crystal‐Induced Inflammation

**DOI:** 10.1111/imm.70108

**Published:** 2026-01-25

**Authors:** Yair Molad, Irina Lagovsky, Vitaly Kliminski

**Affiliations:** ^1^ Laboratory for Research of Inflammation Felsenstein Medical Research Center, Gray's Faculty of Medical and Health Sciences, Tel Aviv University Tel Aviv Israel; ^2^ Institute of Rheumatology, Rabin Medical Center—Beilinson Hospital Petach Tikva Israel

**Keywords:** interleukin‐1 beta (IL‐1β), monosodium urate crystals (MSU), NLRP3 inflammasome, spleen tyrosine kinase (Syk), triggering receptor expressed on myeloid cells‐1 (TREM‐1)

## Abstract

Gout is associated with the upregulation of triggering receptor expressed on myeloid cells‐1 (TREM‐1) and soluble TREM‐1 (sTREM‐1). Cell activation signalling induced by monosodium urate (MSU) crystals and TREM‐1 signalling both converge on spleen tyrosine kinase (Syk). The aim of this study was to decipher the role of the TREM‐1 peptidomimetic inhibitor LP17, as well as Syk inhibitor, and their interaction during MSU‐induced cell inflammation, focusing on NLRP3 inflammasome activation, IL‐1β production and pyroptosis. In MSU‐activated cells, both LP17 and Syk inhibitor (iSyk) significantly reduced the secretion of IL‐1β and the release and activity of caspase‐1. LP17 changed the phosphorylation of Syk, indicating that inhibition of TREM‐1 modulates the activation state of Syk. Both LP17 and iSyk reduced the level of NLRP3‐induced apoptosis‐associated speck‐like protein (ASC) transcripts and MSU‐induced immunolabelling of ASC. Under confocal immunomicroscopy, TREM‐1 in MSU‐crystal‐activated cells was localised to the same perimembranal compartment with NLRP3 inflammasome; inhibition of TREM‐1 hindered the colocalisation of Syk with ASC. These results were corroborated by use of the in vitro ASC oligomerszation assay that showed that blocking TREM‐1 induced Syk redistribution from ASC complexes, indicating that LP17 can repress the ability of Syk to incorporate into the forming ASC speckle. Additionally, unlike iSyk, LP17 hampered MSU‐induced cleavage of gasdermin D, the hallmark of pyroptosis. Together, our findings suggest that blocking TREM‐1 may prove beneficial as a novel strategy in the treatment of gout as well as other inflammasome‐mediated diseases.

## Graphical abstract

Schematic overview of the roles triggering receptor expressed on myeloid cells‐1 (TREM‐1) plays in monosodium urate (MSU) crystal‐induced inflammasome activation and IL‐1β secretion in gout arthritis. The present study provides novel data suggesting that blockade of TREM‐1 by LP17 attenuates MSU‐crystal‐induced inflammation through inhibition of sequential steps in inflammasome activation and IL‐1β secretion: caspase‐1 release and activity, Syk phosphorylation, ASC production and intracellular Syk‐ASC complex formation. In addition, the results showed that LP17 inhibits cleavage of GSDMD to its active form (GSDMD‐N) and monocyte membrane pore formation, leading to reduced IL‐1β secretion.

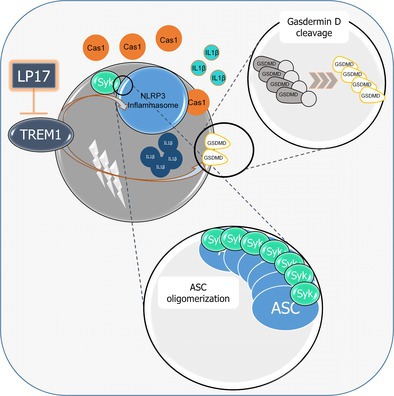

**Abbreviations**
ASC: apoptosis‐associated speck‐like protein
GSDMD: gasdermin
GSDMD‐N: N terminal domain of GSDMD
iSyk: Syk inhibitor
MSU: monosodium urate
NLRP3: nucleotide‐binding and oligomerisation domain (NOD)‐like receptor (NLRs) P3
sTREM‐1: soluble TREM‐1
Syk: spleen tyrosine kinase
TREM‐1: triggering receptor expressed on myeloid cells‐1

## 1 Introduction

Gout arthritis typically resolves spontaneously [1]. An attack of gout is initiated by monocyte/macrophage phagocytosis of monosodium urate (MSU) crystals deposited in joints and cell activation through the NLRP3 inflammasome [2]. This results in the release of IL‐1β and subsequent recruitment of leucocytes into the inflamed joint [3, 4, 5].

Activation of the NLRP3 inflammasome leads to the assembly of a multimeric complex comprised of a pyrin domain and an adaptor molecule called apoptosis‐associated speck‐like protein containing a caspase recruiting domain (ASC). Pro‐caspase‐1 is recruited to ASC, leading to activation of caspase‐1 which mediates the conversion of IL‐1β to its active form. It also cleaves gasdermin D (GSDMD), causing pyroptosis, a type of inflammatory cell death [6, 7].

Triggering receptor expressed on myeloid cells‐1 (TREM‐1) is constitutively expressed on the surface of CD14^+^ monocytes and neutrophils. It is upregulated by Toll‐like receptor (TLR)‐4 ligand LPS and cytokines, and it amplifies the production of pro‐inflammatory cytokines [8, 9, 10, 11]. Soluble TREM‐1 (sTREM‐1) is composed of the extracellular domain of TREM‐1 that is shed from activated myeloid cells [10, 12] and acts as a decoy receptor by sequestering TREM‐1 ligand [12, 13]. TREM‐1 and sTREM‐1 levels were shown to be increased in gout [14, 15, 16]. Moreover, the synthetic peptide LP17, which resembles sTREM‐1 [12], was reported to suppress the release of proinflammatory cytokines.

TREM‐1 signalling requires the adaptor molecule DAP12 [10]. Spleen tyrosine kinase (Syk) plays a crucial role in DAP12 signalling [17] which can be prevented by Syk inhibitor (iSyk) [18]. Syk is a critical regulator of NLRP3 inflammasome activation, exerting its effect through phosphorylation of ASC, which leads to ASC oligomerisation and caspase‐1 activation [19], It is also involved in the downstream pathway of TREM‐1‐DAP12 [10, 20, 21, 22] as well as TREM‐1 coupling with DAP12 [13, 23, 24].

Recently, we have shown in a mouse model of gout that LP17 ameliorates MSU crystal‐induced inflammation by decreasing pro‐inflammatory cytokines and chemokines level with no effect on the level of the anti‐inflammatory cytokine transforming growth factor‐β1 (TGFβ) [25]. The aim of this study was to analyse the effects of LPI7 as well as iSyk on NLRP3 inflammasome activation, IL‐1β production and pyroptosis.

## 2 Materials and Methods

### 2.1 Materials and Reagents

Cell culture media and supplements were purchased from Gibco, USA. Other compounds and reagents used in the study were the Syk‐specific inhibitor ER27319 maleate (Tocris, Bio‐Techne, USA), protease inhibitor cocktail (Mercury, USA), DC protein assay kit (BioRad, USA), Caspase‐1 activity assay (G9951, Promega, USA), 7AAD (eBioscience, Thermo Fisher Scientific, USA) and disuccinimidyl suberate (Cayman Chemical, USA). In addition, Höechst 33342, MSU, lipopolysaccharide (*Escherichia coli*, 055: B5), and 4% paraformaldehyde (PFA) were purchased from Merck (USA). The LP17 peptide corresponding to the developmentally conserved extracellular sequence of membranal TREM‐1 (LQVTDSGLYRCVIYHPP; Genemed Synthesis, USA) or its scrambled sequence variant was used to block TREM‐1.

#### 2.1.1 Antibodies

The list of antibodies used in the article is shown in Supporting Information: Table 2.

### 2.2 Preparation of MSU Crystals

MSU crystals were prepared by crystallisation of a supersaturated solution of uric acid under mildly basic conditions, according to the modified protocol of Martin et al. [26] Details are presented in Data S1. The experiments required 500 μg MSU crystals for monocyte stimulation.

### 2.3 Cell Culture and Experiments

All experiments were performed on THP‐1 human monocytic leukaemia cells (TIB‐202). Details are presented in Data S1.

### 2.4 FACS

For assessment of inflammasome ASC expression in THP‐1 cells we used FACS analysis as described in detail in Data S1. Gating was performed by marking the ASC‐immunolabelled cell population over isotype control rabbit IgG in MSU‐activated THP‐1 cells and application on subsequent samples.

### 2.5 Quantitative Reverse Transcriptase PCR

Quantitative reverse transcriptase PCR (RT‐qPCR) was performed as previously described [26] with the High‐Capacity cDNA Reverse Transcription Kit and Fast SyBR TM Green Master Mix and run on the StepOne Plus machine (Applied Biosystems, ThermoFisher Scientific, USA). The primer sets are presented in the Supporting Information: Table 1.

### 2.6 Protein Extraction/Western Blot Analysis

Western blot was performed as described previously [27]. Detailed methods are described in Data S1.

### 2.7 Immuno‐Confocal Microscopy and Colocalisation Assessment

Cells were collected by centrifugation at 150*g*, fixed in PFA (4%, in PBS), rinsed twice in 0.5% PBS‐BSA, blocked with 5% normal goat serum in PBS, rinsed again and incubated overnight at 4°C with primary anti‐Syk and/or anti‐ASC antibodies diluted with the blocking solution. The next day, cells were rinsed in 0.5% PBS‐BSA and incubated with the appropriate fluorophore‐labelled (568 nm) goat antimouse and/or goat antirabbit (488 nm) antibodies. Cell nuclei were labelled with Höechst, washed twice and mounted on slides using antifading reagent (Dako, Agilent Technologies, USA). Visualisation and image acquisition were performed using the Leica TCS SP8 Confocal Imaging System (Leica Microsystems, Germany) employing the ×20 air objective, and findings were analysed with the Leica Application Suite X. Colocalisation was assessed using ImageJ software (NIH).

### 2.8 ASC Oligomerisation Assay

ASC in vitro oligomerisation assay was performed according to the Methods and Protocols described for NLR proteins by Khare et al. [28] Detailed method is presented in Data S1.

### 2.9 ELISA

The following ELISA kits were used: IL‐1β (PeproTech, USA), Caspase‐1 and soluble TREM‐1 (R&D Systems, Bio‐Techne, USA). The process was performed according to the manufacturer's instructions, with minor changes, as described in Data S1.

### 2.10 Caspase‐1 Activity Assay

This assay was performed using the Caspase‐Glo1 Inflammasome Assay (G9951, Promega, USA) based on a bioluminescent method to directly measure caspase‐1 activity. Briefly, 6 × 10^6^ (62 500/well) cells were seeded on a 96‐well plate (NUNC, Norway) and treated as described previously. The medium and cells were collected, separated and processed according to the manufacturer's assay instructions and references within. Measurements were made every 10 min on a luminometer (Berthold, USA), according to the protocol, for at least 2 h.

### 2.11 Statistical Analysis

Each experiment was performed at least three times. Data are expressed as mean ± SD unless stated otherwise. Statistical analyses were performed using Prism (GraphPad Prism, Dotmatics, USA) with one‐way ANOVA followed by Fisher's least significant difference (LSD) post hoc test (uncorrected). A *p* value of < 0.05 was considered statistically significant.

## 3 Results

### 3.1 Blockade of TREM‐1 by LP17 Ameliorates MSU‐Crystal‐Induced Inflammation

**FIGURE 1:**
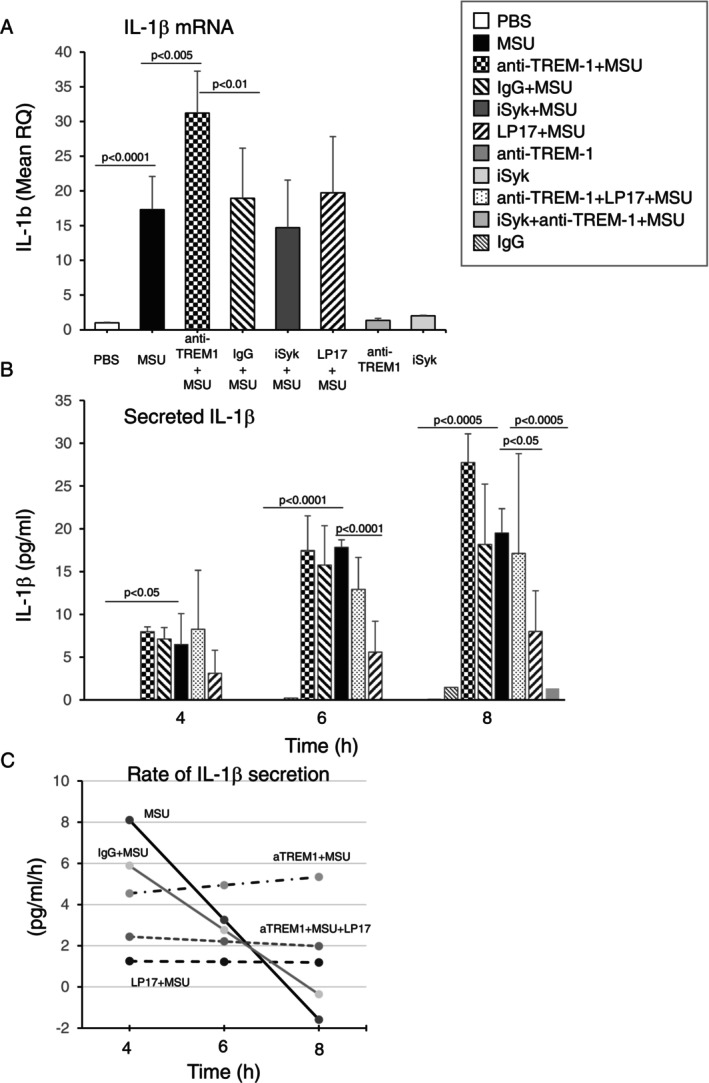
Blockade of TREM‐1 by LP17 and iSyk reduces MSU crystal‐induced IL‐1β production. (A) Level of IL1β transcript in THP1 cells pretreated with anti‐TREM‐1 antibody (anti‐TREM‐1) or isotype control (IgG), Syk inhibitor (iSyk) or LP17 peptide (LP17), harvested after 4 h stimulation with 500 μg MSU or PBS, assayed by comparative RT‐qPCR. Mean RQ + SD of three independent experiments; (B) IL‐1β (pg/mL) secreted from THP1 cells pretreated with anti‐TREM‐1 antibody (anti‐TREM1) or isotype control (IgG), Syk inhibitor (iSyk) or LP17 peptide, detected by ELISA after 4, 6 and 8 h stimulation with 500 μg MSU or PBS. Mean + SD of three independent experiments; (C) Rate of IL‐1β secretion (pg/mL/h) calculated by applying second degree polynomial regression on data presented in 1B and producing the first derivative of the resulted equations. Note the increase in IL‐1β production rate in anti‐TREM‐1 treated samples (aTREM1 + MSU).

The first step was to define the role of TREM‐1 in the production of IL‐1β in MSU‐activated cells; specifically, whether Syk is involved in this process. RT‐qPCR assay showed that in THP‐1 cells stimulated with MSU, the transcript level of *IL‐1β* was high and the effect was further amplified in cells pretreated with agonist anti‐TREM‐1 antibody (Figure 1A). Neither LP17 nor iSyk affected the expression of *IL‐1β* (Figure 1A). Next, we analysed the level of secreted IL‐1β under the same conditions at 4, 6 and 8 h of cell activation with MSU (ELISA). Following treatment with anti‐TREM‐1 antibody, an increase was observed only after 8 h (Figure 1B). Both LP17 and iSyk significantly reduced IL‐1β secretion at 6 and 8 h, after stimulation with MSU alone and on co‐stimulation with agonist anti‐TREM‐1 antibody (Figure 1B).

To determine the effect of LP17 over time in MSU‐activated THP‐1 cells, we calculated the IL‐1β secretion rate under the studied conditions by plotting the equations of IL‐1β secretion (see Figure 1B) against a time scale (Figure 1C). The MSU‐induced IL‐1β secretion rate was high at 4 h and declined gradually thereafter, whereas the level of anti‐TREM‐1 in the presence of MSU gradually increased at 4, 6 and 8 h (Figure 1C). In MSU‐activated cells pretreated with LP17, the rate of IL‐1β secretion remained constantly low (Figure 1C).

Taken together, these results show that anti‐TREM‐1 antibody increases the expression of *IL‐1β mRNA* in MSU‐induced inflammation. The addition of LP17 or iSyk suppresses secretion of the IL‐1β peptide but does not affect the *IL‐1β* transcript.

### 3.2 Blockade of TREM‐1 by LP17 Modifies the Phosphorylation Pattern of Syk

To investigate the effect of TREM‐1 on the activation of Syk, WB was applied to analyse Syk phosphorylation at tyrosine residues 525 and 323, which have been found to be essential for Syk kinase activity and downstream signalling [29]. Protein samples were collected at 5, 15 and 60 min following MSU‐induced THP‐1 cell activation. After 5 min in the presence of LP17 (Figure S1), Syk phosphorylation at the Y525 residue reverted to baseline (PBS) (*p* < 0.05) (Figure 1A,B); no change was detected after 15 and 60 min. LP17 also significantly changed Syk phosphorylation at Y323 at 60 min compared to the effect of MSU alone (*p* < 0.05) (Figure 1C,D).

These results suggest that LP17 alters MSU‐induced Syk phosphorylation.

### 3.3 Blockade of TREM‐1 by LP17 Inhibits Caspase‐1 Activity

**FIGURE 2:**
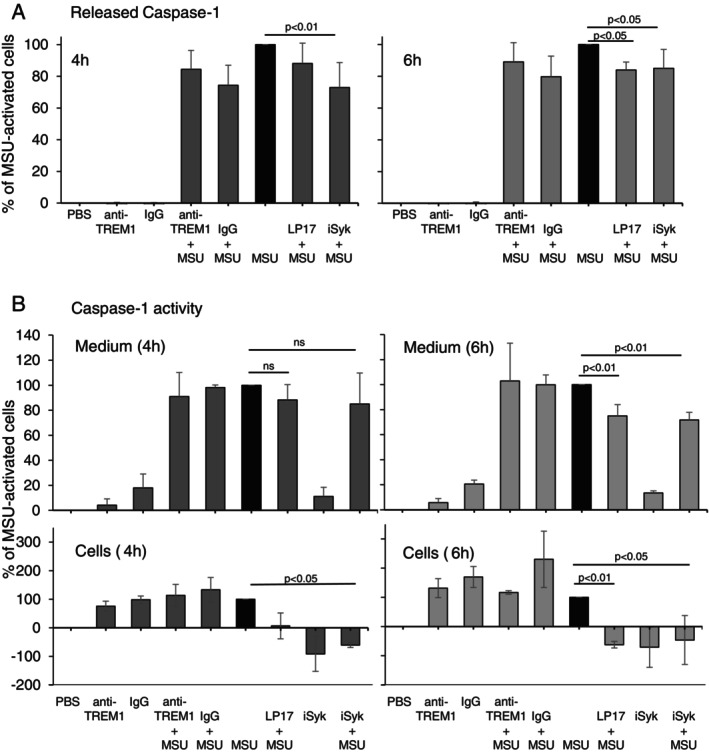
Blockade of TREM‐1 by LP17 and inhibition of Syk decrease MSU crystal‐induced caspase‐1 release and activity. (A) ELISA analysis of Caspase‐1 secreted by THP‐1 cells pretreated with anti‐TREM‐1 antibody (anti‐TREM1) or isotype control (IgG), Syk inhibitor (iSyk) or LP17 peptide, after 4 (left) and 6 h (right) stimulation with 500 μg of MSU. Percentage, mean ± SD of three independent experiments; (B) Caspase‐1 activity assay performed on medium (medium, upper panel) or cell lysate (cells, lower panel) samples of THP‐1 cells pretreated with anti‐TREM1 antibody (anti‐TREM1) or isotype control (IgG), Syk inhibitor (iSyk) or LP17 peptide (LP17) after 4‐ and 6‐h stimulation with 500 μg of MSU. Activity value (%) calculated by subtracting the background and dividing by the value of MSU‐stimulated sample. Mean ± SD of three independent experiments; ns—not significant statistically.

Secretion of active caspase‐1, one of the prominent hallmarks of NLRP3‐inflammasome activity, was assessed (ELISA) after activation of THP‐1 cells with MSU for 4 and 6 h (Figure 2). Stimulation with anti‐TREM‐1 antibody did not have any effect compared with MSU alone. Pretreatment with LP17 significantly decreased caspase‐1 level at 6 h following MSU cell activation, and iSyk had a significant effect at both 4 and 6 h (Figure 2A).

On Caspase‐1 assay study, after 4 h of MSU activation, the extracellular activity of caspase‐1 was unaffected by either treatment compared to MSU (Figure 2B, upper left panel) but its intracellular activity was robustly reduced by iSyk (Figure 2B, low left panel). After 6 h of MSU activation, LP17 and iSyk each significantly reduced extracellular caspase‐1 activity (*p* = 0.008) (Figure 2B, upper right panel); the addition of both LP17 and iSyk completely abolished intracellular caspase‐1 activity (Figure 2B, lower right panel).

These data suggest that blocking TREM‐1 significantly inhibits caspase‐1 activity in MSU‐induced inflammation.

### 3.4 Blockade of TREM‐1 by LP17 Reduces ASC Expression

**FIGURE 3:**
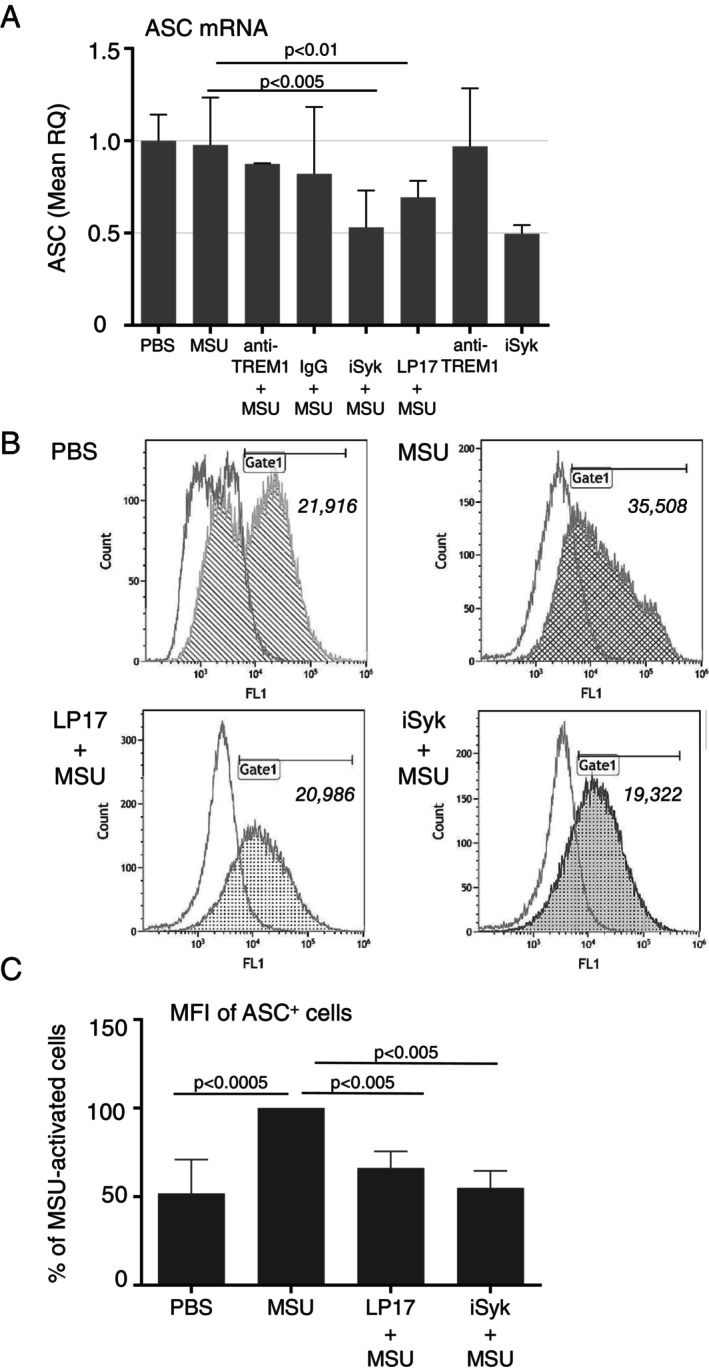
Attenuation of TREM‐1 signalling by LP17 peptide and inhibition of Syk reduce the expression of ASC in response to MSU crystals. (A) Level of ASC transcript in THP‐1 cells pretreated with anti‐TREM‐1 antibody (anti‐TREM‐1) or isotype control (IgG), Syk inhibitor (iSyk) or LP17 peptide, harvested after 4 h of stimulation with 500 μg MSU or PBS, assayed by comparative RT‐qPCR. Mean RQ ± SD of 3 independent experiments. (B) Representative histograms of FACS analysis of intracellular ASC immunolabelling in THP‐1 cells indicated (*italics*) the gated mean fluorescence intensity (MFI) of ASC immunostained cells (filled), over cells incubated with isotype control antibody (clear). Gating performed by marking the ASC‐immunolabelled cell population over isotype control rabbit IgG in MSU‐activated THP‐1 cells and applying on subsequent samples. (C) Analysis of three independent experiments of intracellular ASC immunolabelled cells FACS analyses, normalised to MSU‐activated cells (100%), Mean ± SD.

In view of our finding of suppressed IL‐1β secretion and caspase‐1 activity in LP17‐treated THP‐1 cells, we sought to determine if inhibition of TREM‐1 in MSU‐stimulated cells can regulate the assembly and activation of the NLRP3 inflammasome complex [30]. Although no change was seen in mRNA transcripts of *ASC* following stimulation with MSU crystals only, LP17 as well as iSyk significantly reduced the expression of *ASC* mRNA (by 25% *p* < 0.01 and by 50% *p* < 0.005, respectively; Figure 3A). On FACS analysis, MSU‐induced cell activation was associated with a significant increase in ASC level (*p* < 0.0005), while the addition of LP17 or iSyk significantly reduced MSU‐induced ASC protein expression (*p* < 0.005) (Figure 3B,C).

These results suggest that blocking TREM‐1 as well as iSyk leads to a significant decrease in *ASC mRNA* and ASC protein labelling during MSU‐induced monocyte activation.

### 3.5 Blockade of TREM‐1 by LP17 Reduces Membrane‐Bound Co‐Localisation of Syk With ASC‐Contained Complex

**FIGURE 4:**
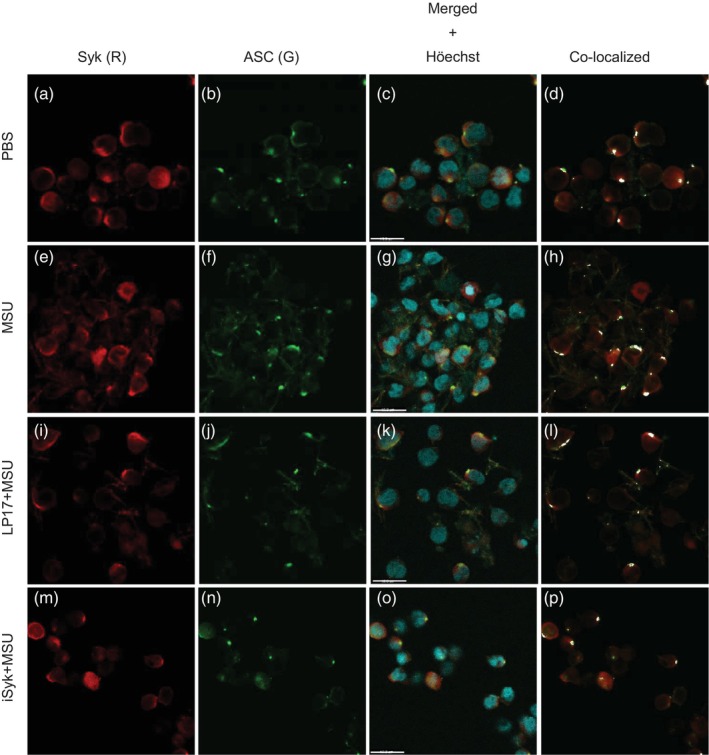
LP17 peptide and inhibition of Syk modify the cellular distribution and co‐localisation of ASC and Syk. Confocal immunomicroscopy images of untreated THP‐1 cells (PBS, a‐d), and MSU‐activated (e‐h), pretreated with either LP17 (LP17+MSU, i‐h) or Syk inhibitor (iSyk+MSU, m‐p), labelled for Syk (red), ASC (green) and nuclei (Höechst dye, blue). The analysis for colocalisation (d, h, l, p, in white) performed using ImageJ software. Note the change in colocalisation pattern in the perimembranal region from dotted in the control cells (d), to streched in MSU‐activated cells (h), a decrease in stretched pattern in LP17‐pretreated cells (l), and almost complete elimination od colocalisation in iSyk‐pretreated cells (p). Representative images of three independent experiments. Scale bar 19.3 μm.

Based on previous reports showing that Syk interacts with ASC and induces ASC oligomerisation [30, 31, 32, 33], we assessed the effect of TREM‐1 inhibition on the cellular distribution and co‐localisation of ASC and Syk in MSU‐treated THP‐1 cells. While the cellular distribution of Syk was partially membranal in the control (PBS) cells, the distribution of ASC had a membranal and punctate pattern (Figure 4) characterised by low ASC‐Syk co‐localisation. MSU stimulation induced ASC redistribution to the cell membrane, leading to its increased co‐localisation with Syk (Figure 4, in white). LP17 partially reduced the extent of ASC‐Syk co‐localisation, whereas iSyk completely restored the punctate membranal distribution pattern of ASC, comparable to that of untreated cells (Figure 4b and Figure S2).

**FIGURE 5:**
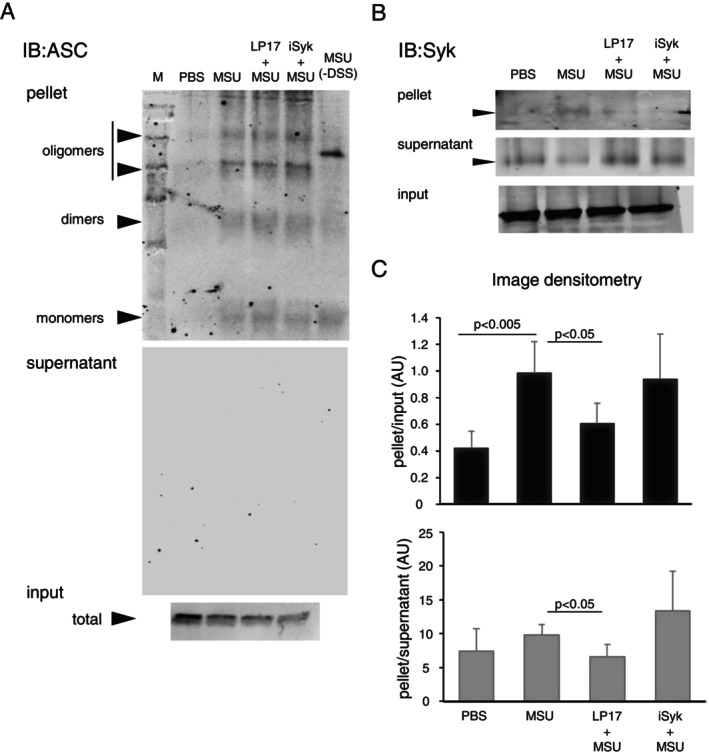
In MSU‐activated cells, blockade of TREM‐1 causes Syk redistribution from ASC containing complexes in vitro. (A) Immunoblot of ASC in vitro oligomerisation assay. MSU‐activated cells pretreated with either LP17 or iSyk, lysed after 6 h and processed, proteins resolved by SDS‐PAGE. *Upper panel*, representative immunoblot of pelleted assay products, arrowheads indicating the retrieved ASC protein species, note their absence in the control MSU sample, processed without the crosslinker (‐DSS). Also note, *middle panel*, the absence of ASC protein species in the immunoblot of the supernatant products of the samples above. Lower panel, input lysates, reserved before the assay, showing total ASC protein in the sample. (B) Syk immunoblot performed on the products of ASC oligomerisation assay, as in A (upper panel). Note higher Syk content in the pellet of MSU‐activated cells, versus lower Syk in the LP17‐pretreated samples. Also note that in the supernatant, low Syk content in MSU‐activated cells versus higher Syk in LP17‐pretreated cells. Representative image of 4 independent experiments. (C) Densitometry analyses of Syk immunoblots of pelleted assay products, normalised to input (upper) or supernatant (lower), mean ± SD of four independent experiments.

To further investigate the effect of TREM‐1 inhibition on NLRP3 inflammasome assembly, we implemented the ASC in vitro oligomerisation assay using DSS‐mediated cross‐linking of cell extracts from MSU‐stimulated THP‐1 cells [19]. Although there was a clear effect of MSU crystals on ASC dimer and multimer assembly (Figure 5A), no significant changes were noted in the ability of ASC to self‐assemble in extracts from LP17 or iSyk‐pretreated cells at either 4 h (data not shown) or 6 h (Figure 5A,B) of MSU stimulation (uncropped images of Figure 5 are presented in Figure S3). Prompted by our observations of ASC and Syk co‐localisation and the suppressive effect of both LP17 and iSyk, we probed the same membranes with an anti‐Syk antibody (Figure 5B). Notably, the ASC‐enriched pellet fraction contained more Syk in MSU‐stimulated cells (Figure 5B) whereas cells pretreated with LP17 contained less Syk, although the input concentration was equal. Moreover, there was an inverse trend in the ASC‐depleted supernatant fraction, with a low content of Syk in the MSU‐stimulated cells and a higher content of Syk in the LP17‐pretreated cells. Further analysis of Syk distribution normalised to input (Figure 5C) and of pellet‐to‐supernatant ratio corroborated the effect of LP17 on the intracellular redistribution of Syk to the sites of ASC assembly and NLRP3 complex formation in the MSU‐stimulated cells.

Taken together, these findings show that LP17 prevents the redistribution of Syk to sites of ASC speck assembly in MSU‐activated monocytes.

### 3.6 Blockade of TREM‐1 by LP17 Reduces Pyroptosis

**FIGURE 6:**
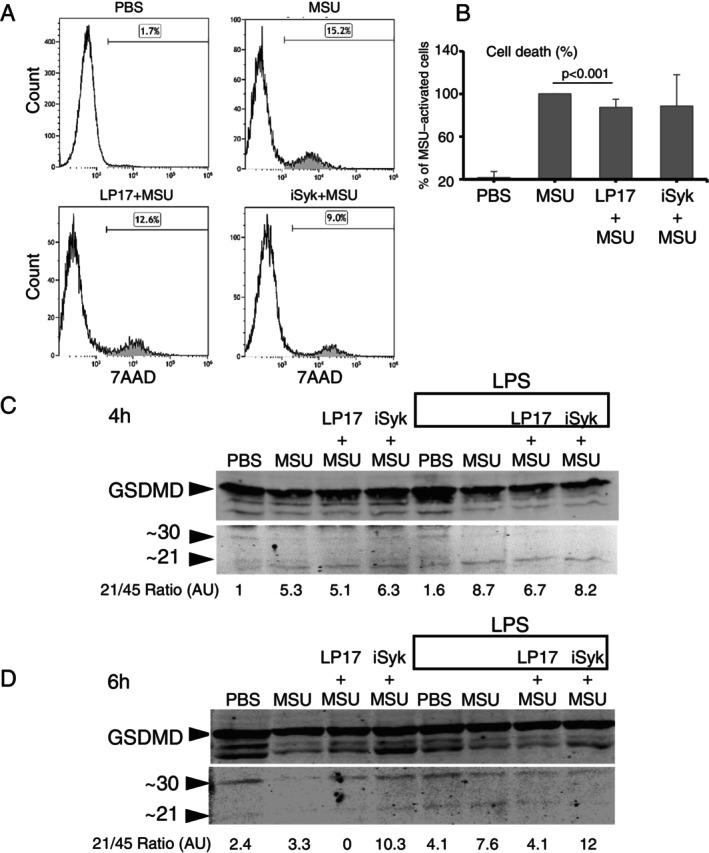
Blockade of TREM1 reduces pyroptosis in cells activated with MSU crystals. (A) Representative flow cytometry analysis of intracellular accumulation of 7AAD in THP‐1 cells, pretreated with LP17 or iSyk, following 4 h of activation with MSU; shaded area showing the dead cells, the percentage indicated above. (B) Combined analysis of five independent experiments of intracellular 7AAD accumulation in THP1 cells, normalised to MSU activated cells (100%), Mean ± SD. (C and D) Representative immunoblot showing Gasdermin D (GSDMD) in cells pretreated with LP17 or iSyk, with or w/o LPS, lysed 4 (C) and 6 h (D) after activation with MSU; proteins resolved by SDS‐PAGE, arrowheads indicate the retrieved GSDMD protein species. Numbers correspond to densitometry analysis of cleaved (~21 kDa) to noncleaved (~45 kDa) species ratio. Note the increased cleavage of Gasdermin D in cells pretreated with LPS.

Cleavage of the N‐terminal domain of GSDMD (GSDMD‐N) has been found to mediate the release of IL‐1β via membrane pores [34]. FACS analysis of 7AAD vital dye incorporation was used to assess the effect of LP17 on pyroptosis in MSU‐stimulated cells, untreated or pretreated with LP17 or iSyk (Figure 6A,B). Whereas MSU‐induced THP‐1 activation increased 7AAD dye incorporation, LP17 pretreatment significantly reduced the number of stained cells (*p* < 0.01). Addition of iSyk was not associated with a significant change compared with MSU alone.

To corroborate these findings, we assessed proteolytic pyroptosis‐mediated GSDMD cleavage. MSU stimulation of THP‐1 cells for 4 h increased the ratio of GSDMD‐N (21 kD) to uncleaved GSDMD (45 kD) compared to PBS‐treated control cells (Figure 6C). This effect was amplified by cell pretreatment with LPS. The effect of either LP17 or iSyk at 4 h was negligible, but in LPS‐activated cells, LP17 had a slight inhibitory effect. At 6 h of MSU stimulation, pretreatment with LP17 peptide completely abolished GSDMD cleavage whereas iSyk had an inverse effect (Figure 6C,D). After 6 h of MSU stimulation, LPS‐activated cells showed the same GSDMD cleavage pattern as after 4 h.

These results suggest that blocking TREM‐1 inhibits caspase‐1‐mediated GSDMD cleavage to its active form, leading to decreased IL‐1β secretion in MSU‐induced inflammation.

## 4 Discussion

Gout arthritis usually subsides spontaneously [1, 2]. The present study provides novel data suggesting that LP17 attenuates MSU‐crystal‐induced inflammation through inhibition of sequential steps in inflammasome activation and IL‐1β secretion: caspase‐1 release and activity, Syk phosphorylation, ASC production and intracellular Syk‐ASC complex formation. In addition, the results showed that LP17 inhibited cleavage of GSDMD to its active form (GSDMD‐N) and monocyte membrane pore formation, leading to reduced IL‐1β secretion. We also present data showing the effect of Syk inhibition on inflammasome assembly and caspase‐1 activation, comparable to, although more potent than the effect of LP17. However, Syk inhibition did not reduce caspase‐1‐mediated GSDMD‐N formation and pyroptosis. Thus, while TREM‐1 apparently plays a pivotal role in NLRP3 assembly and caspase‐1 activation as well as in GSDMD‐N‐mediated pyroptosis, Syk is involved in MSU‐induced inflammasome activity and regulation of IL‐1β production, but not in GSDMD‐N formation.

Phagocytosis of MSU crystals serves as a signal for activation of the NLRP3 inflammasome, resulting in the release of IL‐1β [2, 3]. Consistent with previous studies [8, 9, 10], we showed that monocyte stimulation with MSU crystals induced a marked increase in *IL‐1β* mRNA, and membrane TREM‐1 ligation with an agonist anti‐TREM‐1 antibody significantly amplified late *IL‐1β* production (Figure 1). Furthermore, LP17 significantly suppressed monocyte IL‐1β release, but it did not inhibit *IL‐1β* mRNA. These results suggest that membrane TREM‐1 binding amplifies the stimulatory effect of MSU crystals, and that LP17 suppresses MSU‐induced IL‐1β production.

IL‐1β is a key mediator of the MSU‐induced inflammatory response [31]. Following ASC recruitment to the NLRP3 inflammasome, activated caspase‐1 mediates IL‐1β processing from its inactive precursor state to its active form [6, 7]. In the present study, MSU crystals induced an increase in the level and activity of caspase‐1; agonist anti‐TREM‐1 antibody did not have an additive effect. Blockade of monocyte TREM‐1 by LP17 significantly decreased caspase‐1 level and activity following MSU crystal stimulation (Figure 2), implying that TREM‐1 is involved in downstream signalling of MSU crystal stimulation and plays a role in the initiation of gout arthritis flare.

Upon NLRP3 activation, NLRP3 and ASC relocate to form a perinuclear punctate structure (‘speck’) that is crucial for the inflammasome response [19, 33]. ASC oligomerisation is a hallmark of inflammasome activation [33]. Therefore, we sought to determine whether LP17 inhibits ASC oligomerisation in response to MSU crystals. We showed, for the first time, that blocking TREM‐1 suppressed NLRP3 inflammasome activation via suppression of ASC oligomerisation (Figure 4). Moreover, our unpublished observation shows that upon MSU‐induced cell activation, TREM‐1 co‐localised with NLRP3 as well as with ASC (Figure 4a) within the same membranal region where NLRP3 co‐localised and ASC (data not shown).

Studies have clearly shown that the oligomerisation of ASC is dependent on Syk, which can phosphorylate ASC on Tyr 146 and Tyr 186 [33]. We found that under LP17‐mediated inactivation of TREM‐1, Syk exhibited low incorporation into the forming ASC oligomer. The same pattern in the ASC‐containing ‘specks’ was demonstrated by immunostaining LP17‐pretreated, MSU‐stimulated THP‐1 cells. Inhibiting TREM‐1 led to lesser assembly of ASC complexes (Figure 3B,C) and lowered the processivity of the NLRP3 complex, suggested by the analysis of IL‐1β secretion rate. Thus, we postulate that while TREM‐1 activation, by promoting phosphorylation of Syk, is important for the assembly of the NLRP3 inflammasome, TREM‐1 inhibition prevents the incorporation of Syk into ASC oligomers, hindering the processivity of the NLRP3 complex.

Caspase‐1 activation leads to GSDMD‐N‐mediated membrane pore formation, a pivotal step in pyroptosis [34]. Herein we demonstrate that TREM‐1 blockade using LP17 reduced GSDMD‐N formation and pyroptosis (Figure 6).

Earlier reports suggested that activation of the NLRP3 inflammasome upregulates macrophage TREM‐1 expression [35]. Our study is the first to demonstrate the suppressive effects of TREM‐1 inhibition on NLRP3 inflammasome activation in MSU‐activated monocytes, thereby shedding new light on the role of TREM‐1 in the process of IL‐1β production in gout.

Syk has been shown to contribute to NLRP3 inflammasome‐mediated caspase‐1 activation [19, 30] and was found to be critical in signalling MSU‐induced macrophage production of IL‐1β [19]. Our data demonstrated that Syk inhibition significantly suppressed monocyte IL‐1β production in addition to caspase‐1 protein level and activity (Figures 1 and 2), ASC expression (Figure 3), and membrane‐bound co‐localisation of Syk with ASC (Figure 4). Unlike LP17, Syk inhibition did not affect either GSDMD‐N formation or pyroptosis. In summary, NLRP3 assembly, caspase‐1 activation and IL‐β secretion were significantly suppressed by Syk inhibitor treatment, implicating Syk as an upstream regulator of MSU‐crystal activation signalling and TREM‐1‐mediated signalling pathways in gout.

To avoid a positive feedback loop of cell activation and IL‐1β release in gout arthritis, stringent control of these processes is essential. Previous studies have shown that TGFβ [36] as well as neutrophil extracellular traps promote the spontaneous resolution of MSU‐crystal‐induced arthritis [37]. We have recently shown in a mouse model of gout that LP7 selectively suppresses pro‐inflammatory cytokines and chemokine, with no significant effect on TGFβ level leading to diminished recruitment of leucocytes into the inflamed site [25]. Our study adds novel data suggesting that TREM‐1 plays a role in inflammasome activation and pyroptosis and that sTREM‐1 plays an essential role in spontaneous resolution of gout attack.

In conclusion, blocking TREM‐1 may prove beneficial as a novel strategy for the treatment of gout as well as other inflammasome‐mediated autoinflammatory diseases.

## Author Contributions

Y.M. and V.K. contributed to the conception and design of the work; analysis and interpretation of data for the work and drafting, reviewing and final approval of the version to be published. I.L. and V.K. performed experiments, and I.L. contributed to the analysis of the data and contributed to the writing of the manuscript.

## Funding

The authors have nothing to report.

## Ethics Statement

The study did not involve experiments on human tissue or cells or on animals. This study did not require ethics approval by the IRB of Rabin Medical Centre and/or Felsenstein Medical Research Centre.

## Conflicts of Interest

The authors declare no conflicts of interest.

## Supporting information


**Data S1:** imm70108‐sup‐0001‐Supinfo1.docx.

**Figure S1:** Blockade of TREM‐1 modulates MSU crystal‐induced phosphorylation state of Syk in THP1 cells. (A) Representative immunoblots of phospho‐(Y525) and total‐Syk in THP‐1 cells pretreated with either LP17 or PBS, collected and lysed at 5‐, 15‐ and 60‐min of activation with MSU crystals. (B) Combined densitometry analyses, calculated as pY525‐Syk to total‐Syk ratio, mean ± SD of three independent experiments, *p* < 0.05. (C) Representative immunoblots of phospho‐ (Y323) and total‐Syk in THP1 cells pretreated with either LP17 or PBS, collected and lysed at 5‐,15‐ and 60‐min of activation with MSU crystals. (D) Combined densitometry analysis, calculated as pY323‐Syk to total‐Syk ratio, mean ± SD of three independent experiments, *p* < 0.05. Note the increased phosphorylation of Y323 residue in MSU‐activated cells along the time‐course, significantly reduced by LP17 following 60 min of MSU‐activation.

**Figure S2:** LP17 peptide and inhibition of Syk modify the cellular distribution and co‐localisation of ASC and Syk. Quantification of ASC and Syk co‐localisation indices of corresponding treatments generated by ImageJ software using a co‐localisation plug‐in. Presented are values normalised to MSU values. Mean ± SD of three experiments, *p* < 0.05.

**Figure S3:** The uncropped version of immunoblot images presented in Figure 5. The outlined boxes correspond to the cropped image areas of the immunoblots presented in Figure 5A,B.

## Data Availability

All data supporting the results reported in this article are included in the article and its Supporting Information: Data S1. Further enquiries can be directed to the corresponding author (Y.M., contact details: ymolad@tauex.tau.ac.il) upon reasonable request.
