# Supplementary material for: TREM‐1 Blockade Inhibits Inflammasome Activation and Pyroptosis: Novel Insights on the Role of TREM‐1 and Syk in Monosodium Urate Crystal‐Induced Inflammation
Source: Immunology. 2026 Jan 25;178(2):249–60. doi: 10.1111/imm.70108 (PMC13135878; doi:10.1111/imm.70108)
Supplement: Supplementary file 1 — Data S1: imm70108‐sup‐0001‐Supinfo1.docx. [file IMM-178-249-s001.docx]

**Supplement**

**Methods**

**Preparation of MSU crystals (**Martin et al.^25^ **)**

Briefly, 2 grams of uric acid was added to 1L sterile 1M NaOH solution and boiled until fully dissolved. The pH was adjusted to 7.2 with 1N HCl, and the solution gradually cooled to room temperature overnight, while stirring. The next day, the sedimented crystals were passed through sterile mesh, washed with ethanol and acetone, and dried at 45ºC overnight under sterile conditions. The resulting needle-shaped MSU crystals were inspected under brightfield and polarized light microscopy to verify their size and birefringence. The crystals were subjected to Limulus amoebocyte cell lysate assay (ToxinSensor Chromogenic LAL Endotoxin Assay Kit, GenScipt, USA) and found to be endotoxin-free (<0.01 EU). (EU-Endotoxin Unit).

**Cell culture and experiments**

THP-1 human monocytic leukemia cells (TIB-202^TM^) were obtained from the American Type Culture Collection (ATCC, USA) and maintained in RPMI1640 supplemented with 10% FBS, 100units/ml penicillin, 100μg/ml streptomycin, and 1mM L-glutamine (Gibco, USA). For MSU-induced activation, cells were seeded at 1*10^6^ cells/ml in 35mm plates (Nunclon^TM^, Thermo, USA). The next day, before treatment with MSU crystals (333 µg/ml), the following compounds were added: LP17 (0.1µg/ml for 30 minutes), Syk (3.125µM for 20 minutes), or anti-TREM-1 (4µg/ml for 90 min), same as isotype control IgG LPS (100ng/ml O/N).

**Table Suppl. 1: Primers used in real-time PCR**

| **mRNA** | **FWD** | **REV** |
| --- | --- | --- |
| RPLPO | TCGTGGAAGTGACATCGTCTTT | CTGTCTTCCCTGGGCATCA |
| IL1β | GAGCACCTTCTTTCCCTTCA | TCGTGCACATAAGCCTGGTTA |
| ASC | CTCACCGCTAACGTGCTGC | GATAAGCGCAGCCCGGTG |

**Protein extraction/western blot analysis**^26^

Briefly, cells were collected, centrifuged at 150xg, washed with ice-cold PBS, lysed in lysis buffer (10% SDS, 20% glycerol, 120mM Tris-HCl, pH 6.8), and boiled for 10 minutes, and aliquots were removed to determine protein concentration. Thereafter β-mercaptanol-supplemented Laemmli's sample buffer was added followed by repeated boiling for 5 minutes. Proteins were resolved on 12-15% polyacrylamide-SDS gels (30-40µg/lane), transferred onto nitrocellulose membranes (Novex, ThermoFisher Scientific, USA), and immunoblotted with target-specific antibodies. Reactive bands were detected and quantified with the Odyssey^®^ Infrared Imaging System (Li-Cor Biotechnology, USA) using goat anti-rabbit IgG, IRDye800 (Rockland Technologies, USA) and goat anti-mouse, DyLight680 (Invitrogen, USA) fluorophore-labeled antibodies.

**Table Suppl. 2:** The list of antibodies:

| Target | Manufacturer | Cat# | Application (dilution) |
| --- | --- | --- | --- |
| anti-ASC | AbCam, USA  Santa Cruz, USA | ab155970  F9, sc-271054 | ASC *in-vitro* oligomerization assay (1:100), Flow Cytometry (1:50); Confocal immuno-microscopy (1:50)  IB (1:500) |
| anti-Syk | Santa Cruz, USA | sc-1240 | confocal immuno-microscopy (1:50), IB (1:500) |
| anti-pY323-Syk | Sigma-Aldrich, USA | SAB4300282 | IB (1:100) |
| anti-pY525-Syk | Sigma-Aldrich, USA | SAB4503839 | IB (1:100) |
| anti-GSDM-D | Cell Signaling Technology, USA | E83GF | IB (1:500) |
| anti-TREM-1 | BioLegend, USA | 316102 | *in-vitro* cell activation (4µg/ml) |
| anti-rabbit isotype control | AbCam, USA | AB172730 | Flow Cytometry (1µL per 1.0x10^6^ cells) |
| Trustain FcX | BioLegend, USA | 422301 | Flow Cytometry |

**ASC oligomerization assay**^27^

Briefly, 3x10^6^ THP-1 cells were harvested, centrifuged at 400*g for 5 minutes, lysed, and sheared by passing 10 times through a 21-gauge needle in 0.5ml of ASC lysis buffer (20mM HEPES–KOH, pH 7.5, 150mM KCl, 1% NP-40, 0.1mM PMSF, and 1mM sodium orthovanadate, complemented with protease inhibitor cocktail). The input was reserved, and the remaining lysate centrifuged at 2500xg for 10 minutes. The pellet was resuspended in 0.5ml PBS supplemented with 2mM DSS and incubated at room temperature for 45 minutes on a rotator. The reaction was then centrifuged at 2500xg, and the cross-linking reaction was quenched by resuspension in 30µL of Laemmli's sample buffer and boiling for 10 minutes at 95°C. The proteins were resolved by running on 15% SDS-PAGE along with the appropriate reserved input samples, transferred on the nitrocellulose membrane, and immunoblotted for the detection of ASC dimers, multimers, and Syk.

**Flow cytometry (FACS)**

THP-1 cells were blocked with anti-Fc receptor antibody (FcX), incubated at 4°C for 40 minutes with anti-TREM1 antibody. For inflammasome ASC labeling, cells were fixed in 4% PFA for 20 minutes on ice, washed with 0.5% PBS-BSA and blocked with FcX for 10 minutes on ice and incubated, first with an anti-ASC antibody and then with a second anti-rabbit FITC conjugated antibody, washed with PBS, and analyzed by FACS (Beckman Coulter, USA) using Kaluza software (Beckman Coulter, USA).

**Enzyme-linked immunosorbent assay (ELISA)**

Conditioned media were collected and centrifuged at 1000xg for 5 minutes to obtain cell-free supernatants and stored at -80°C (except IL-1β samples which were processed immediately).

The assay was performed according to the manufacturer's instructions, with minor changes: The immunosorbent plates (Corning, USA) were coated overnight at 4°C, washed and blocked, and samples were incubated overnight at 4°C and equilibrated at room temperature for 2 hours. All measurements were performed in duplicate and analyzed on an ELISA reader (Biotech, USA) after wavelength correction (450nm vs. 570nm readings) using 4 parametric logistic (4-PL) curve fit analyses.
