# Supplementary figures and images for: TREM‐1 Blockade Inhibits Inflammasome Activation and Pyroptosis: Novel Insights on the Role of TREM‐1 and Syk in Monosodium Urate Crystal‐Induced Inflammation
Source: Immunology. 2026 Jan 25;178(2):249–60. doi: 10.1111/imm.70108 (PMC13135878; doi:10.1111/imm.70108)

Suppl. Figure 1

A

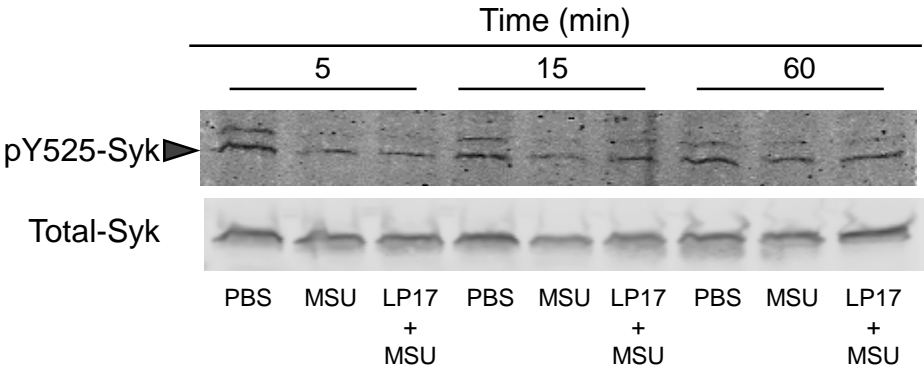

B

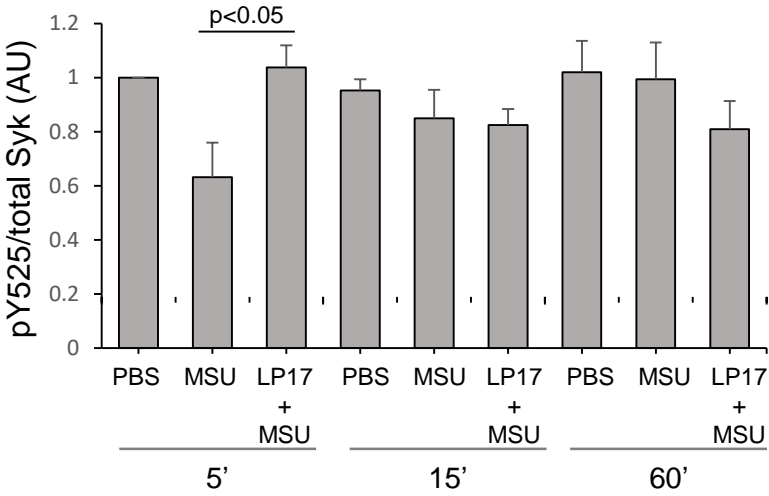

C

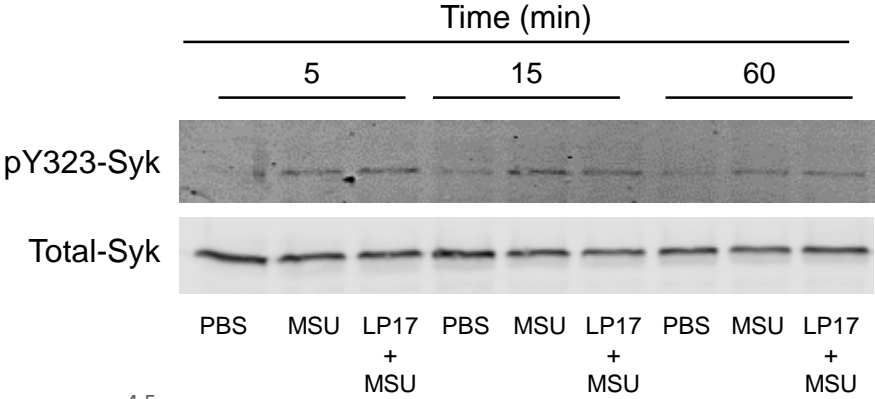

D

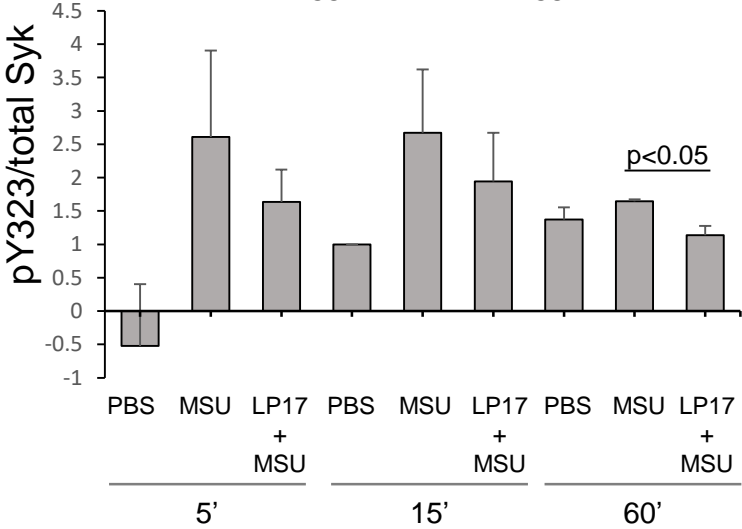

Supplement: Supplementary file 2 — Figure S1: Blockade of TREM‐1 modulates MSU crystal‐induced phosphorylation state of Syk in THP1 cells. (A) Representative immunoblots of phospho‐(Y525) and total‐Syk in THP‐1 cells pretreated with either LP17 or PBS, collected and lysed at 5‐, 15‐ and 60‐min of activation with MSU crystals. (B) Combined densitometry analyses, calculated as pY525‐Syk to total‐Syk ratio, mean ± SD of three independent experiments, p < 0.05. (C) Representative immunoblots of phospho‐ (Y323) and total‐Syk in THP1 cells pretreated with either LP17 or PBS, collected and lysed at 5‐,15‐ and 60‐min of activation with MSU crystals. (D) Combined densitometry analysis, calculated as pY323‐Syk to total‐Syk ratio, mean ± SD of three independent experiments, p < 0.05. Note the increased phosphorylation of Y323 residue in MSU‐activated cells along the time‐course, significantly reduced by LP17 following 60 min of MSU‐activation. [file IMM-178-249-s002.pdf]

Suppl. Figure 2

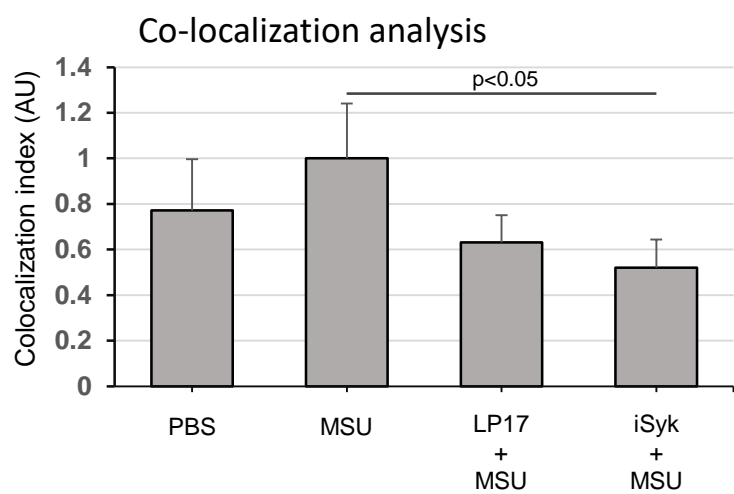

Supplement: Supplementary file 3 — Figure S2: LP17 peptide and inhibition of Syk modify the cellular distribution and co‐localisation of ASC and Syk. Quantification of ASC and Syk co‐localisation indices of corresponding treatments generated by ImageJ software using a co‐localisation plug‐in. Presented are values normalised to MSU values. Mean ± SD of three experiments, p < 0.05. [file IMM-178-249-s003.pdf]

Suppl. Figure 3

A

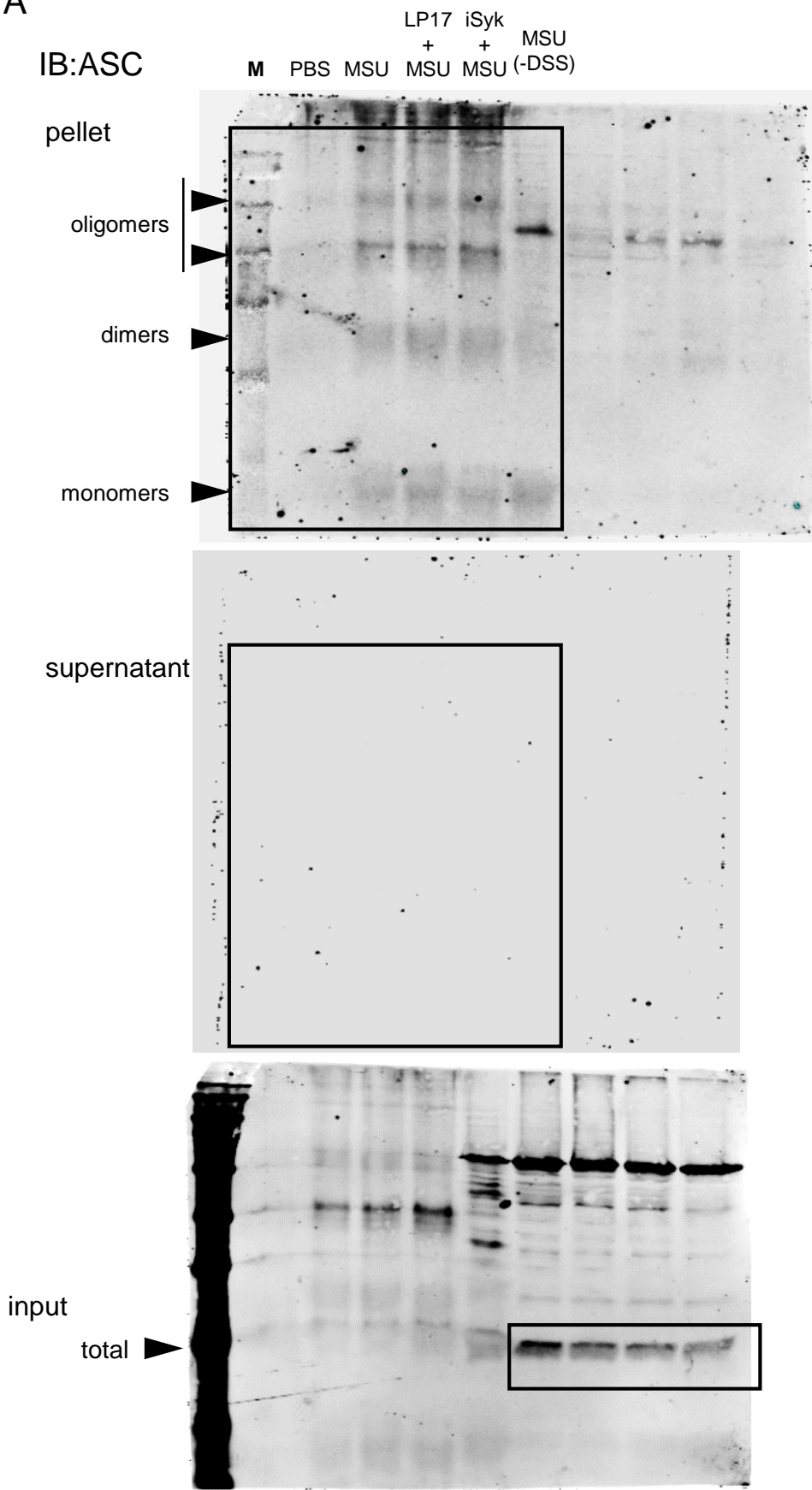

B

IB:Syk

PBS    MSU    LP17  
              +  
              MSU    iSyk  
                      +  
                      MSU

pellet

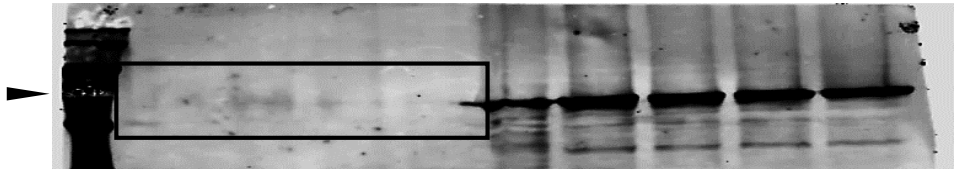

supernatant

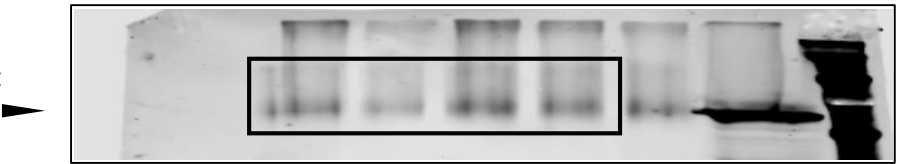

input

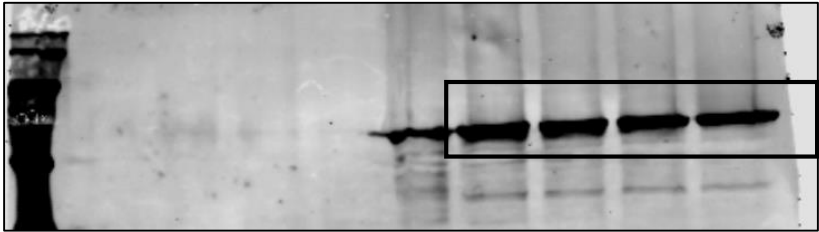

Supplement: Supplementary file 4 — Figure S3: The uncropped version of immunoblot images presented in Figure 5. The outlined boxes correspond to the cropped image areas of the immunoblots presented in Figure 5A,B. [file IMM-178-249-s004.pdf]
